# Effects of endozoochory and diploendozoochory by captive wild mammals on *Juniperus deppeana* seeds

**DOI:** 10.1002/ece3.10262

**Published:** 2023-07-04

**Authors:** Fabián Alejandro Rubalcava‐Castillo, Arturo Gerardo Valdivia‐Flores, José de Jesús Luna‐Ruíz, Luis Ignacio Íñiguez‐Dávalos, Víctor Manuel Martínez‐Calderón, Antonio de Jesús Meraz Jiménez, Joaquín Sosa‐Ramírez

**Affiliations:** ^1^ Centro de Ciencias Agropecuarias Universidad Autónoma de Aguascalientes Aguascalientes Mexico; ^2^ Departamento de Ecología y Recursos Naturales, Centro Universitario de la Costa Sur Universidad de Guadalajara Autlán de Navarro Mexico

**Keywords:** carnivores, diploendozoochory, mammals in captivity, seed dispersal

## Abstract

Carnivorous mammals disperse seeds through endozoochory and diploendozoochory. The former consists of ingestion of the fruit, passage through the digestive tract, and expulsion of the seeds, a process that allows scarification and dispersal of the seeds over long or short distances. The latter is typical of predators that expel seeds that were contained in the prey and the effects of which may differ from those of endozoochory with respect to the retention time of the seeds in the tracts, as well as their scarification and viability. The objective of this study was to conduct an experimental evaluation comparing the capacity of each mammal species in terms of the dispersal of *Juniperus deppeana* seeds and, at the same time, to compare this capacity through the two dispersal systems: endozoochory and diploendozoochory. We measured dispersal capacity using indices of recovery, viability, changes in testas, and retention time of seeds in the digestive tract. *Juniperus deppeana* fruits were collected in the Sierra Fría Protected Natural Area in Aguascalientes, Mexico, and were administered in the diet of captive mammals: gray fox (*Urocyon cinereoargenteus*), coati (*Nasua narica*) and domestic rabbits (*Oryctolagus cuniculus*). These three mammals represented the endozoochoric dispersers. For the diploendozoochoric treatment, seeds excreted by rabbits were incorporated into the diets of captive mammals: bobcat (*Lynx rufus*) and cougar (*Puma concolor*), in a local zoo. Seeds present in the scats were then collected, and recovery rates and retention times were estimated. Viability was estimated by X‐ray optical densitometry and testa thicknesses were measured and surfaces checked using scanning electron microscopy. The results showed a recovery of seeds greater than 70% in all the animals. The retention time was <24 h in the endozoochory but longer at 24–96 h in the diploendozoochory (*p* < .05). Seed viability ($\bar{x}$ ± SD) was decreased in rabbits (74.0 ± 11.5%), compared to fruits obtained directly from the canopy (89.7 ± 2.0%), while gray fox, coati, bobcat, and cougar did not affect seed viability (*p* < .05). An increase in the thickness of the testas was also observed in seeds excreted from all mammals (*p* < .05). Through evaluation, our results suggest that mammalian endozoochory and diploendozoochory contribute to the dispersal of *J. deppeana* by maintaining viable seeds with adaptive characteristics in the testa to promote forest regeneration and restoration. In particular, feline predators can provide an ecosystem service through scarification and seed dispersal.

## Graphical abstract

We conducted an evaluation of seed dispersal in mammals: gray fox, coati, rabbit, bobcat, and cougar. We found high recovery percentages and retention times of seeds in the digestive tracts. Seeds remained viable and showed an increase in testes thickness. Our results suggest that endozoochoric and diploendozoochoric mammals contribute to dispersal potential by maintaining viable seeds with adaptive characteristics in the testa to promote resilience and forest restoration.

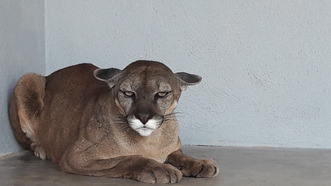

## 1 INTRODUCTION

Endozoochory is the system by which animals disperse seeds through the direct consumption of fruits. The seeds contained in the fruits pass through the digestive tract to subsequently be defecated or regurgitated and dispersed (Cypher & Cypher, 1999; Kleyheeg & van Leeuwen, 2015). In this context, terrestrial mammals such as rabbits (Lezama‐Delgado et al., 2016; Malo & Suárez, 1995), foxes (Escribano‐Ávila et al., 2014), and even larger herbivores (Campos‐Arceiz & Blake, 2011) act as the dispersers of different fruit species. Frugivorous animals are, therefore, essential dispersers of plants (Montiel & Montaña, 2000). However, most of the studies that have evaluated dispersal by endozoochory have focused on small flying vertebrates, such as birds or bats (Aziz et al., 2021; Silva et al., 2021) or larger vertebrates such as primates (Chapman & Dunham, 2018) and even elephants (Poulsen et al., 2021) and many gaps remain in the knowledge of seed dispersal, particularity in carnivorous mammals.

Some groups of mammals, such as frugivores and herbivores, can consume large quantities of fruits and keep the seeds in their digestive tract for long periods, up to several days in some cases (Pedrosa et al., 2019; Poulsen et al., 2021; Rubalcava‐Castillo et al., 2021). In the case of some species of mammals with large ranges of movement, the seeds can be dispersed several kilometers from the parent plant (Cousens et al., 2010; Cypher & Cypher, 1999). These plant‐disperser interactions are essential for the dynamics of plant communities (Levin et al., 2003; Levine & Murrell, 2003), especially in settings where environmental disturbance and changes have taken place (Lundberg & Moberg, 2003; Montoya et al., 2008).

The passage of seeds through the digestive tract of the animal is a critical phase in endozoochory because the survival of seeds in the digestive system is one of the main determinants of the success of endozoochorous dispersal. Complex interactions are, therefore, established between the characteristics of the animals and plants for their survival in the digestive tract (Cosyns et al., 2005; Stiegler et al., 2021). To understand how mammalian handling and gut passage affect seeds; for example, viability and scarification, it is essential to conduct seed‐feeding experiments under controlled experimental conditions. However, it is important to take into consideration that the variability that exists between plants and animals means that seed‐feeding experiments can present contrasting results, with greater germination success found in some cases (Grande et al., 2013; Mancilla‐Leytón et al., 2011) and lower success in others (D'hondt & Hoffmann, 2011; Grande et al., 2013). The potential for endozoochorous dispersal in many plant species has been evaluated using seed‐feeding experiments, particularly with herbivores (Poulsen et al., 2021; Sadeghayobi et al., 2011; Wang et al., 2017) such as domestic rabbits, sheep, and cows (Cosyns et al., 2005) or by the germination testing of collected scat samples (Milotić & Hoffmann, 2016; Salinas & Reynoso, 2023; Stiegler et al., 2021).

Diploendozoochory is a complex process involving the participation of two or more dispersing agents in sequence (prey and predator). Specifically, this type of dispersal occurs when a carnivorous predator consumes a primary disperser or seed predator, along with the seeds in the digestive tract of the prey, and subsequently deposits the seeds along with the remains of the prey in feces or regurgitated pellets (Nogales et al., 2007, 2012). Unlike conventional endozoochory in which the seed passes through only one digestive tract of the primary disperser, the participation of a carnivore in the second phase of the diploendozoochory process can influence the plant dispersal in three ways: (1) the transport of seeds, since the distribution areas of predators are usually wider, (2) the alteration of their viability, since the seed is in contact with the gastric acids of two digestive tracts and (3) the change in the abundance of dispersed seeds, because the seeds dispersed by the carnivore will depend on the number of seeds present within the prey at the time of ingestion (Hämäläinen et al., 2017). Based on the above, the existing studies have focused exclusively on identifying the species involved and determining the number of seeds dispersed as well as the estimated dispersal distance. One investigation in this area, carried out by Sarasola et al. (2016), studied the cougar (*Puma concolor*), which, over a long distance, dispersed large numbers of seeds of herbaceous species originally consumed by its prey, the dove (*Zenaida auriculata*). Likewise, Nogales et al. (2007), found in scats from birds of prey of the species *Lanius meridionalis* and *Falco tinnunculus* that there were three species of seeds associated with the remains of lizards (*Gallotia atlantica*); suggesting that these two predatory birds could disperse these plants by diploendozoochory through the incidental ingestion of seeds that were contained inside the lizards. On the other hand, Reiserer et al. (2018) showed that rattlesnakes of the genus Crotalus consumed heteromids with seeds in their cheek pouches, and by examining the entire intestine, they also discovered that secondarily ingested seeds could germinate in the colon of the snake, demonstrating the importance of this group of vertebrates in the rescue and dispersal of seeds.

Recently, Rubalcava‐Castillo et al. (2021) documented diploendozoochory in *Juniperus deppeana* seeds dispersed by bobcats (*Lynx rufus*) through their prey, the rabbit (*Sylvilagus* sp.). The juniper or táscate (*Juniperus deppeana* Steud; Cupressaceae) has a fruit that is adapted to dispersal by endozoochory and diploendozoochory (Rubalcava‐Castillo et al., 2020). It is a pioneer species of wide distribution that colonizes post‐disturbance areas and is vital to the recovery of forests of the Sierra Fría Protected Natural Area (SF‐PNA) in Aguascalientes, Mexico (Díaz‐Núñez et al., 2016). One of the factors associated with the wide distribution of the juniper is its seed dispersal through terrestrial mammals (Rubalcava‐Castillo et al., 2020). The passage of seeds through the digestive tract of mammals does not affect their viability so they can be dispersed without complications (Rubalcava‐Castillo et al., 2021).

Studies of diploendozoochory have shown that strictly carnivorous predators, such as felines, could play a role in the ecosystem like that of a seed disperser. However, research related to the understanding of diploendozoochory in cats is scarce, and no information is available to explain the comparable efficiency of diploendozoochory and endozoochory, for example, through a feeding experiment. Thus, seed‐feeding experiments are essential to determine the adaptive characteristics, such as resistance to stomach acid and its effect on the surface of the testa (Venier et al., 2012), and the time that seeds remain in the digestive tracts of dispersers. These variables critically influence seed survival during and after passage through the intestine (Tsuji et al., 2020). Evaluation of such seed survival enables determination of the dispersal capacities of the different plant species involved (Costea et al., 2016; Tsuji et al., 2020; Venier et al., 2012).

Since most of this research has focused on medium‐sized mammals, such as the fox and the marten (Graae et al., 2004; Tsuji et al., 2020), we studied three mammals that perform endozoochory: gray fox, coati, and rabbit. In addition, felines were incorporated into the evaluation to demonstrate the participation of these large mammals in seed dispersal through the process of diploendozoochory (Rubalcava‐Castillo et al., 2020; Sarasola et al., 2016; Tsuji et al., 2011). The objective of this study was to evaluate the capacity of *Juniperus deppeana* seeds for dispersal by mammals in an endozoochory and diploendozoochory feeding experiment, using indices of recovery, viability, changes in testas, and retention of seeds in the digestive tract. Specifically, we wished to demonstrate whether there is a difference between the influence of endozoochory by frugivores (gray fox, coati, and rabbit) and that of diploendozoochory by hypercarnivores (bobcat and cougar) on seed dispersal efficiency (number of seeds recovered after ingestion), seed retention time, and seed viability. We also wished to determine whether the thickness of the testa of dispersed seeds decreases and influences seed survival following passage through the intestine. This experimental evaluation is focused on determining the beneficial and detrimental effects experienced by the seed when dispersed by both dispersal systems by examining the effects of different animal species. This system enables the determination of the dispersal capabilities of this plant species through mammal zoochory.

## 2 MATERIALS AND METHODS

An overview of the experimental evaluation is given in this first section, which comprises two main stages, and the particular details of each stage are presented separately below.

The experimental evaluation was conducted in March 2019 and consisted of two stages: (i) to create the habit in mammals of ingesting *J. deppeana* seeds and (ii) the experimental evaluation of endozoochory and diploendozoochory. Prior to the two stages, the collection of seeded fruits of the plant component was carried out. The *J. deppeana* shrub produces globules that generally contain five seeds each, and the fruits of this species are consumed and dispersed by both birds and mammals (Livingston, 1972; Schupp et al., 1997). The animal component was also selected according to the dispersal system: gray fox, coati (omnivores), and rabbit (herbivore/feline prey) for endozoochory, and bobcat and cougar (predators) for diploendozoochory. It is documented that all selected mammals disperse seeds by either one system or the other. The selection of mammals was based on animal species (the gray fox, coati, and bobcat), where the dispersal of *J. deppeana* seeds by endozoochory and diploendozoochory had previously been verified in the forests of the SF‐PNA (Rubalcava‐Castillo et al., 2021). It had also been verified that rabbits can disperse *Juniperus* seeds by endozoochory in forests (Lezama‐Delgado et al., 2016).

The stage I lasted 10 days with the objective of adapting the animals to the ingestion of the fruits and seeds. After 10 days, the rabbits became accustomed to the fruit intake, as did all the other mammals to the seed intake, since the seed rations were eaten completely without any avoidance behavior. Once the animals had adapted, stage II began with the modeling, in which the animals were again offered seeds in their diets for 21 days. During the second stage, the seed retention time in the tracts was estimated by counting the time from seed ingestion to the time of excretion with seeds present where, in turn, the seeds were separated from the scats, collected, and counted. Subsequently, the scat‐derived seeds and those collected from the canopy (control) were subjected to X‐ray and SEM tests.

### 2.1 Control group

A control group was established to test the effects of endozoochory and diploendozoochory in seeds from mammals under evaluation versus seeds taken directly from the tree. During the spring of 2019, according to the calendar of the northern hemisphere meteorological stations, ripe fruits with seeds were collected from twelve random individual *J. deppeana* trees of basal diameter >5 cm and height >2 m. The trees selected according to these criteria corresponded to adult plants with a high probability of bearing ripe fruits (Rubalcava‐Castillo et al., 2020). The fruits were collected when they presented a reddish‐brown color in temperate forests of the SF‐PNA. The collection was carried out in an area of 527 ha, within a temperate forest with a temperate subhumid climate and summer rainfall (Rzedowski, 1978), with an average annual precipitation of 650 mm (SEDESO, 1995). These same trees were also sampled for the collection of fruits with seeds for use in the feeding experiment.

### 2.2 Mammal selection

Seventeen domestic rabbits (*Oryctolagus cuniculus*; California breed, young adults, no detectable morphological or physiological alterations, five males and 12 females were chosen) were used to represent the primary disperser (prey) in the diploendozoochory process. The rabbits were kept in cages 1.60 m in width and 50 cm in height. In the morning, they were first fed with fruits and then with their usual diet consisting of a special mixture of alfalfa and standard commercial food.

The mammals were selected from species in captivity at the Center for Environmental, Cultural, and Recreational Education of the Rodolfo Landeros Park in Aguascalientes City, Mexico. The selected species were females: a gray fox (*Urocyon cinereoargenteus*), a coati (*Nasua narica*), two bobcats, and a cougar. In the case of felines, it has been previously shown that the rabbit forms part of the diet of both the bobcat (Rubalcava‐Castillo et al., 2020) and the cougar (de la Torre & de la Riva, 2009; Monroy‐Vilchis et al., 2009) in the region.

The animals were kept in zoo accommodations that were specially designed to meet the standards of space and habitability, in which they were fed their usual diet with seeds incorporated. The gray fox diet comprised fruits (papaya and melon) and chicken; the coati diet contained fruits (banana, papaya, watermelon, and melon) and chicken. The bobcat diet consisted only of chicken, and the cougar diet included veal and chicken.

#### 2.2.1 Creating the habit of ingesting *J. deppeana* seeds in mammals

As part of the experimental evaluation, all mammals, including the rabbits, underwent a 10‐day adaptation period for the ingestion of *J. deppeana* seeds. In the first part, the rabbits only were offered three whole fruits (equivalent to 15 seeds) daily in the morning to each of the rabbits to familiarize them with their taste, appearance, smell, and consumption. The rabbits were monitored to observe their behavior and any effects of the offered fruits.

In the second part, at the same time as the rabbits, the gray fox, coati, and felines also underwent an adaptation period of eating the seeds. Only the seeds were offered without the whole fruit because these mammals in captivity have pre‐established diets assigned by the institution (melon, banana, and watermelon pieces for gray fox and coati, and chicken breast for bobcat and cougar). The seeds were, therefore, placed within the usual diet of mammals. Small pieces of the fruits were broken for gray foxes and coatis, and pieces of chicken were provided for felines. Small pieces of fruit and chicken with the seeds inside were cut to enable the mammals to ingest the seeded portions in one bite to guarantee the intake of all the seeds and avoid choking problems. Five seeds (1.2 cm average diameter) were placed on each small piece of fruit and chicken (the seeds were inserted into each piece of chicken breast, making sure that the seeds were fixed inside the piece). Three pieces daily were offered to each mammal, and the animals were monitored to ensure they swallowed the food completely and consumed the whole ration.

#### 2.2.2 Experimental evaluation of endozoochory and diploendozoochory

The evaluation consisted of two main parts: (1) endozoochory and (2) diploendozoochory. For endozoochory, seeds were offered to the gray foxes, coatis, and rabbits. Rabbits, in addition to carrying out the endozoochory as the primary disperser of *Juniperus* (Lezama‐Delgado et al., 2016), also represent the prey of felines in diploendozoochory. Therefore, the diploendozoochory was divided into two stages: rabbits and felines. Part of the seeds dispersed by rabbits was used to analyze their endozoochory, and the other part was offered to felines to complete the transit of the seeds through both digestive tracts (prey and predator).

Following the adaptation period, a period of 4 days (96 h) was allowed to elapse to ensure that all seeds from the adaptation period had been defecated. Subsequently, for the experimental evaluation, the same procedure was repeated for the ingestion of seeds in all mammals for 21 days. When the seeds were offered, the exact time of ingestion was recorded. After ingestion, inspections were carried out in the rabbit cages and habitats of other mammals at 1‐h intervals to sweep and collect the scats present. All scats were collected from each habitat of each animal species over the entire plausible retention period, employing an exhaustive search of the entire area of the habitats, which had bare soil rendering visible all the scats deposited in that period. Thus, we ensured that the proportion of recovered seeds was an accurate reflection of the seeds lost to digestion rather than to the habitat. This monitoring was carried out continuously for 12 h, during which visits were made to the habitats to check for the presence of scats or to observe whether the animal was defecating.

Upon finding scats with seeds, the time of discovery was recorded to estimate the retention period in the mammalian tract, and the scats were placed in paper sacks. Until verifying the discovery of all seeds (entire or broken) of the ration in the scats of each species, the corresponding ration of seeds was offered to each one the following day. The scats were washed through a sieve system to extract the seeds, which were then dried and counted to estimate the percentage of recovered seeds for each of the different retention times for each animal species.

For the second stage with felines, the seeds from the pellets were incorporated into the felines' usual diet. The exact times of ingestion and excretion were recorded. In the same way, every hour, visits were made to the habitats of each feline for 10–15 min to check for scats and collect them if present. In this way, it was possible to complete the diploendozoochory system by obtaining in the scats of the felines the seeds that had initially passed through the digestive tract of the rabbits and then through the felines themselves. All the seeds found in the scats of all mammals were stored in brown paper bags to prevent moisture accumulation for subsequent use after 10 days in viability tests and the SEM analysis.

### 2.3 Viability test

The viability test used the control seeds and those from the scats in optical densitometry analysis using X‐ray equipment (Faxitron X‐Ray Corporation, at 10 s and 26 kv intensity), according to the technique proposed by De La Garza and Nepamuceno (1986). The technique consists of observing the radiograph and distinguishing viable seeds with intact testas and embryos from nonviable seeds by the presence of underdeveloped or incomplete embryos and empty seeds or absence of embryos. The seeds from the scats were stored in brown paper bags for 10 days and then a sample of 90 seeds per mammal species was randomly taken for analysis in the X‐ray equipment. Seeds were placed on sheets of acetate paper inside the equipment to determine the percentage values of viable and nonviable seeds based on the density of the radiographs. A densitometric analysis was conducted on each control seed and on those dispersed by each mammal, based on the technique proposed by Rubalcava‐Castillo et al. (2021).

### 2.4 Wear on testa thickness

From the same samples per mammal and control used in the viability test, after 10 days of storage in brown paper bags, two seeds (*n* = 2) per animal species and control were randomly selected for processing and analysis in the SEM. The thickness of the seed testas was analyzed, and the measurements of the control group were compared with those extracted from the scats to determine if wear and other changes in the surface of the testas occur in the same way or whether there are differences between seeds subjected to either endozoochory or diploendozoochory. Whole seeds were used and cut in a sagittal shape. They were then covered with yellow gold (4 min) in a Denton Vacuum apparatus (JFC‐1100®, JEOL LTD). Once prepared, the seeds were placed inside the SEM chamber (JSM‐35C®, JEOL LTD; Dykstra & Reuss, 2003).

To analyze the wear on the seed testas produced by the passage through the digestive tract of mammals, the technique proposed by Rubalcava‐Castillo et al. (2021) was used. This technique consists of observing the seed inside the chamber to take thickness measurements in three parts of the testa with a 40× magnification: (1) the micropyle part, (2) the central part, and (3) the part opposite to the micropyle. Three measurements were taken for each part. In addition, the qualitative characteristics of the surface and interior of the testas were recorded, including any loss of superficial vegetal layers and the presence of holes and cracks in the external and internal parts of the testas.

### 2.5 Statistical analysis

The average percentages of viable seeds recovered from the scats of the entire set of species were compared between the two dispersal systems using the *t*‐Student test for independent observations. In addition, an analysis of variance (ANOVA) and a Tukey's honest significant difference (Tukey's HSD) test were performed to compare the averages values of recovered seeds, retention times among the taxonomic groups (herbivore = rabbit; omnivore = gray fox and coati; carnivore = bobcat and cougar) and testa thickness between each species of mammal, with a confidence level of 95% for both tests, where means a–b with a different literal in each variable presented statistical differences. The analyses were performed using statistical software (Statgraphics, V. 16.1, 2012) with the average values ($\bar{x}$ ± SD) expressed in the response variables as % of seeds recovered/% viability/thickness of testa (μm ± SD), and the explanatory variables as dispersal system (endozoochory and diploendozoochory) and each mammal species. In addition, for the average values of the percentages of viability and thickness by SEM, a Dunnett's test was carried out at 95% confidence to determine the incidence of significant differences between the averages of the percentages of viability and thickness of the seeds extracted from the scats of each mammal and those of the canopy (control).

### 2.6 Ethics regulations for the use of animals in research

The management of all the animal species used in this evaluation was carried out in compliance with the provisions established in the Ethics Regulations for the Use of Animals in Teaching and Research at the Autonomous University of Aguascalientes (UAA, 2019), Code: DI‐PL ‐NO‐37, since the behavior and habitual handling of the animals was not altered.

## 3 RESULTS

### 3.1 Experimental evaluation of endozoochory and diploendozoochory

**FIGURE 1:**
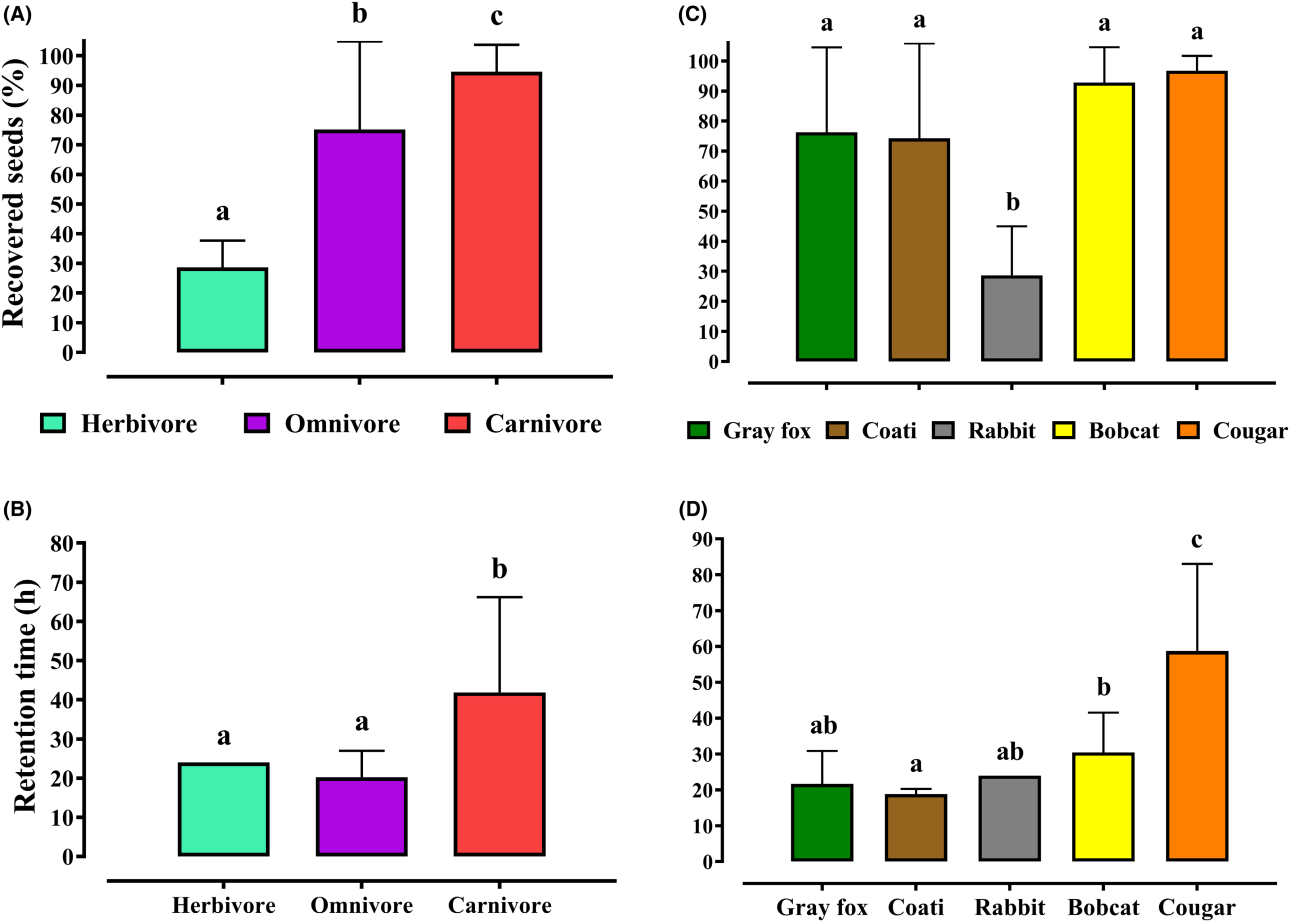
(A) Average percentages ($\bar{x}$ ± SD) of *Juniperus deppeana* seed recovery and (B) average retention times (h) of these seeds in the digestive tract of the taxonomic groups: herbivore = rabbit; omnivore = gray fox and coati; carnivore = bobcat and cougar. (C) Average percentages of seed recovery and (D) mean retention times (h) in the digestive tract of each mammal species. The lines above the bars in the four subfigures indicate the SD. ^a–b^Averages with different literals present statistically significant differences according to the *t*‐Student test (*p* < .05) for the dispersion systems: endozoochory and diploendozoochory, and the Tukey's HSD test (*p* < .05) for the different species of mammals.

Mean seed recovery differed statistically among the different taxa according to their feeding type (*n* = 88, *F* = 26.9, l. g. = 2, *p* < .01). In the carnivores, almost 100% of the seeds offered to diploendozoochoric mammals were recovered in the scats (94.7 ± 9.0%), different from endozoochoric herbivores (28.7 ± 16.3%; Figure 1A). Regarding retention times, there were statistical differences among taxa (*n* = 88, *F* = 22.1, l. g. = 2, *p* < .01) since the carnivores presented a longer mean seed retention time in the feline digestive tract (41.9 ± 23.4 h) in contrast to omnivores (20.2 ± 6.7 h) and herbivores (24.0 ± 0 h; Figure 1B).

Seed recovery exhibited significant differences among the mammals (*n* = 88, *F*_4,84_ = 13.19, *p* < .01). Rabbits (28.7 ± 16.3%) exhibited the lowest recovery percentage because most of the ingested seeds were destroyed by mastication since many crushed seed were found in the pellets. The endozoochorous mammals gray fox (76.2 ± 28.3%) and coati (74.2 ± 31.6%) presented a lower recovery than the diploendozoochorous mammals bobcat (92.8 ± 11.8%) and cougar (96.7 ± 5.0%; Figure 1C).

Seed retention times among mammals differed significantly (*n* = 88, *F*_4,84_ = 30.53, *p* < .01). The diploendozoochorous bobcat (30.4 ± 11.1 h) and cougar (58.7 ± 24.3 h) presented longer retention times than the endozoochorous gray foxes (21.7 ± 9.2 h), coatis (18.8 ± 1.46 h), and rabbits (24 ± 0.01 h; Figure 1D).

### 3.2 Viability

No significant differences were found (*p* > .05) according to Dunnett's paired test when comparing the average viability values of the control against those of the treatments. The seeds sourced from the dispersers did not present significantly higher percentages than those taken directly from the canopy (87.0 ± 5.0%) and the animals do not affect viability either, except for the seeds sourced from the rabbits (74.0 ± 11.5%), the percentage of which was the lowest found during the analysis. Seeds found in the scats of bobcat and cougar maintained the highest mean viability percentage despite being exposed to acids from two digestive tracts (Table 1).

**TABLE 1:** Average viability percentages ($\bar{x}$ ± SD) by X‐ray optical densitometry of seeds from the scats of mammals in captivity (gray fox, coati, rabbits, bobcat, and cougar) compared to the seeds of canopy of the species *Juniperus deppeana*.

Disperser species	Seeds (*n*)	Viability (%)
Directly taken from the canopy (control)	90	87.0 ± 5.0*
Gray fox	90	88.2 ± 21.2*
Coati	90	90.3 ± 11.6*
Rabbit	90	74.0 ± 11.5*
Bobcat	90	91.9 ± 4.7*
Cougar	90	90.9 ± 16.2*

* There were no statistically significant differences according to Dunnett's test (*p* < .05).

### 3.3 Wear on testa thickness

**FIGURE 2:**
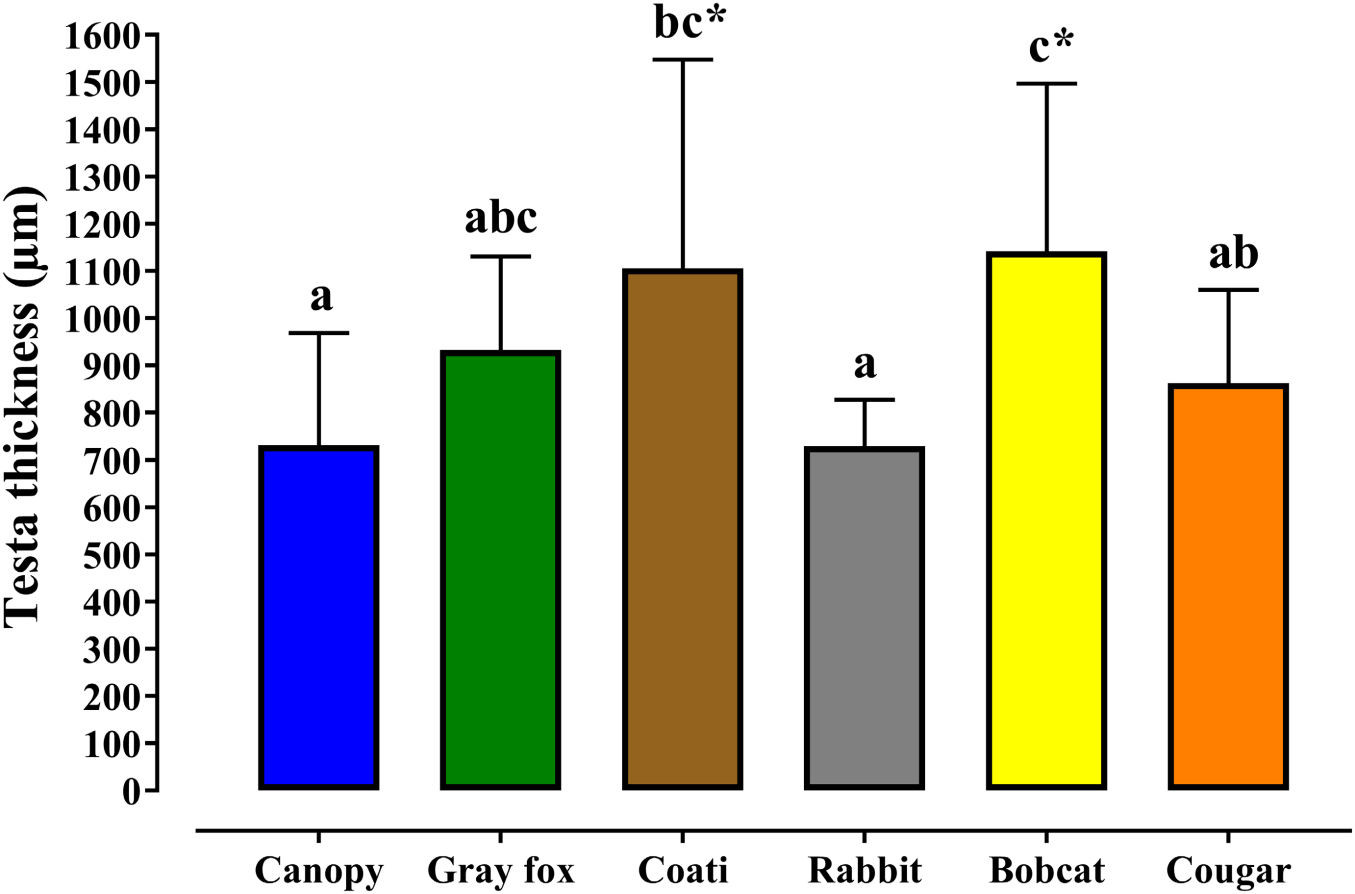
Measurement of the average thickness ($\bar{x}$ ± SD) of *Juniperus deppeana* seed testas from endozoochorous and diploendozoochorous mammal dispersers and directly from the canopy using SEM. ^a–c^Averages with different literals present statistically significant differences according to the Tukey's HSD test (*p* < .05) or * Dunnett's test (*p* < .05).

According to Dunnett's test, significant differences (*p* < .05) were observed between the average thickness of the testa in seeds sourced from the mammals and those of the control. Seeds testas from all mammals, except those excreted by the rabbits (729 ± 98.0 μm), were on average thicker than those of the canopy (731 ± 238 μm), particularly for coati (1106 ± 442 μm) and bobcat (1142 ± 355 μm; Figure 2). In addition, the ANOVA test indicated that there were significant differences between the mean values of each treatment (*F*_5,81_ = 5.46, *p* < .01), verifying that the testa thickness in the seeds excreted by each animal is different.

Dunnett's test showed that the seeds dispersed by the coati (1106 ± 442 μm) and the bobcat (1317 ± 238 μm), with 24 h of retention, presented significant differences compared to those of the canopy. Moreover, those seeds with the longest retention time in the cougars at 72 h (1002 ± 167 μm) and 96 h (937 ± 117 μm) showed a significant increase in the thickness of the testas compared to the control (Table 2).

**TABLE 2:** Average SEM measurements of the thickness ($\bar{x}$ ± SD) of the seed testas of *Juniperus deppeana* from each of the mammals (endozoochory and diploendozoochory) compared to the control group (canopy) and the retention time for each animal species.

Dispersion type	Diet	Species	Seeds (*n*)	Retention time (h)	Seeds (*n*)	Thickness (μm)
Directly taken from the tree (Control)	NA	*J. deppeana*	NA	NA	2	731 ± 238
Endozoochory	Omnivore	Gray fox	319	≤24	2	932 ± 199
		Coati	322		2	1101 ± 442*
	Herbivore	Rabbit	559		2	729 ± 98.0
Diploendozoochory	Carnivore	Bobcat	154	≤24	2	1317 ± 238*
			13	38	2	966 ± 376
		Cougar	9	≤24	2	806 ± 232
			49	48	2	685 ± 123
			10	72	2	1002 ± 167*
			19	96	2	937 ± 117*

* Statistically significant differences with the Control according to Dunnett's test (*p* < .05).

**FIGURE 3:**
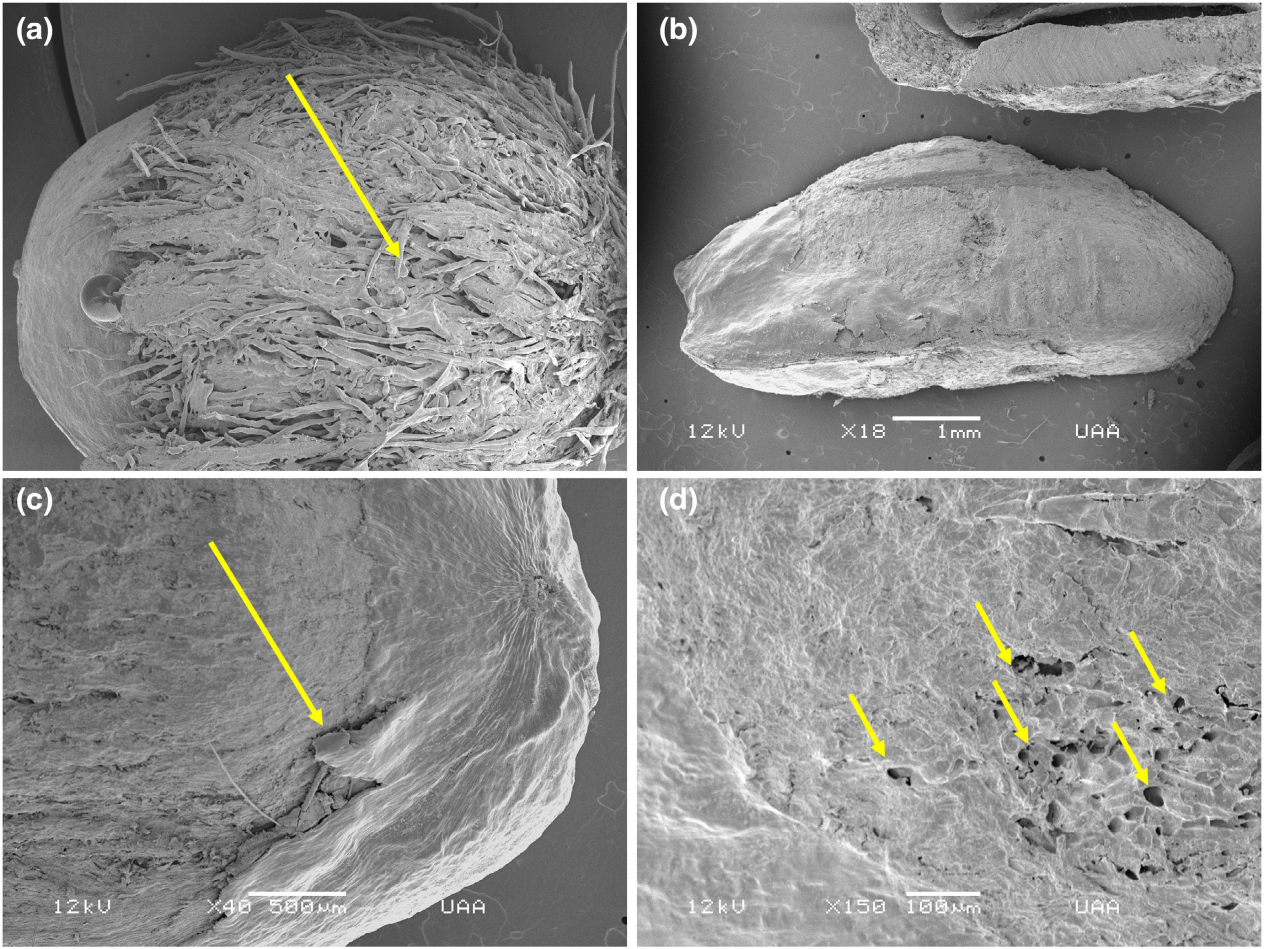
*Juniperus deppeana* seeds taken from the canopy (control) and the scats of mammals (endozoochory). (a) Control seed, with the presence of an external fibrous layer that protects the seed. (b) Seed dispersed by rabbits at 24 h, showing the removal of the outer layer. (c) Seed dispersed by gray fox at 24 h, showing the detachment of a second layer on the surface of the testa. (d) Seed dispersed by the coati at 24 h with testa presenting holes.

Furthermore, it was observed that the seeds taken directly from the canopy presented a hard protective external layer (Figure 3a) that is removed when it passes through the digestive tract of mammals, for example, in the seeds dispersed by rabbits at 24 h (Figure 3b). Wear of a second protective external layer was observed, for example, in the seeds dispersed by the gray fox at 24 h (Figure 3c), and it was even possible to observe the presence of holes in the testa of the seeds from the coati at 24 h (Figure 3d).

**FIGURE 4:**
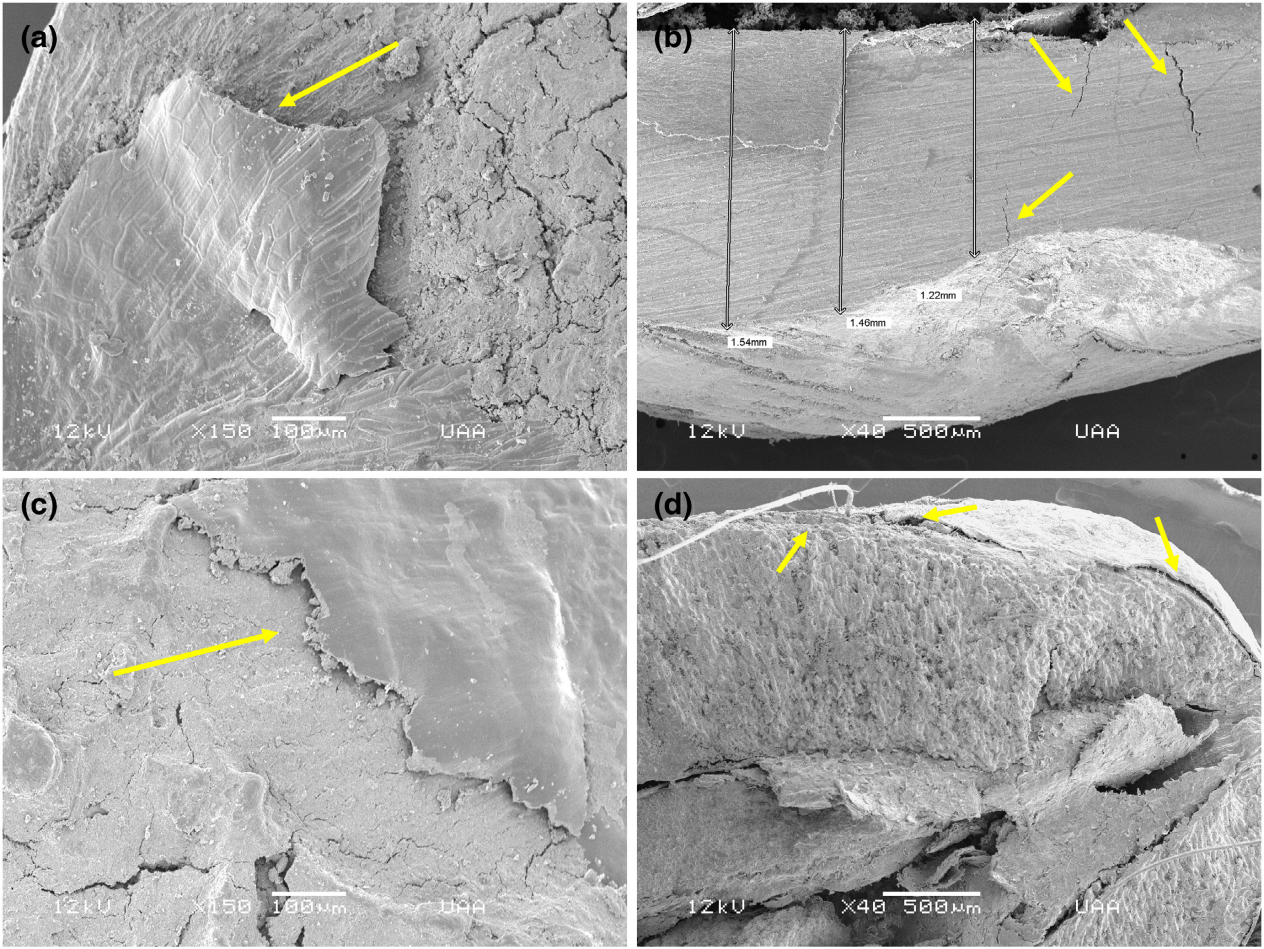
*Juniperus deppeana* seeds taken from the canopy (control) and the scats of mammals (diploendozoochory). (a) Seed dispersed by the bobcat at 24 h, showing removal of the outer layer. (b) Seed dispersed by the bobcat at 24 h, showing cracks inside the testa. (c) Seed dispersed by the cougar at 48 h, showing detachment of the protective external layers of the testa. (d) Seed dispersed by the cougar at 72 h, showing detachment of the protective external layers of the testa and permeability through the width of the testa.

In the diploendozoochory treatment, it was observed in the testa of the seeds from the bobcat at 24 h that both protective external layers were removed (Figure 4a), while there were cracks in the inner part that could have allowed the entry of water into the interior (Figure 4b). In the cougar, it was also possible to appreciate the removal of the protective external plant layer and the superficial layer of the testa at 48 h (Figure 4c). At 72 h, the change in the surface of the testa was more marked (removal of the testa surface), and because it had undergone a longer retention time, a degree of permeability began to be observed (Figure 4d) since there was a formation of small spaces between the testa structures that could allow transfer of water into the interior of the testa.

## 4 DISCUSSION

The evaluation conducted in the present study demonstrated the important participation of local mammals: fox and coati (endozoochory), and bobcat and cougar (diploendozoochory), in the dispersal of *J. deppeana* seeds. This potential for seed dissemination was evidenced by the high percentage of recovered and viable seeds. In turn, our results demonstrated the effect of the first disperser (rabbit) as part of the diploendozoochory process.

Our results showed that seed recovery from the gray fox and coati was relatively high, at more than 70%. Our results support the results in previous models incorporating these types of animals, including that of Graae et al. (2004), who obtained a recovery of 75% in most of the plant species dispersed by the arctic fox (*V. lagopus*), and Varela and Bucher (2006), who obtained more than 84% seed recovery in most of the plant species dispersed by South American foxes (*Lycalopex gymnocercus* and *Cerdocyon thous*). It is, therefore, concluded that the gray fox and the coati are efficient dispersers since most of the seeds they ate were dispersed, and those that were not expelled were possibly destroyed by the mechanical action of chewing or could not be found in the soil of the habitats (ingestion of all the seeds that were offered in the diet of the animals was confirmed).

When beginning the diploendozoochory process in the primary disperser or predator's prey, the rabbit, we found a considerable decrease in the recovery of seeds contained in the pellets because of destruction through intense chewing (Cosyns et al., 2005) of most of the seeds at the time of offering the fruits for ingestion. We thus found a decrease in the number of seeds eliminated through the primary disperser, and the primary disperser, therefore, limits the number of seeds that the secondary disperser can disseminate. Dispersal of seeds by predators thus depends on the number of seeds that remain alive and intact in the digestive tracts of their prey. The second part of the diploendozoochory involving bobcat and cougar predators presented good results by recovering more than 90% of the seeds, which makes the strictly carnivorous mammals in this study efficient seed dispersers. Although there was a significant reduction in the number of seeds during their passage through the first disperser, it can be concluded that at least 90% of the seeds that remain in the prey will be disseminated by the secondary disperser. Consequently, secondary dispersal by carnivores could have important implications for the functioning of disturbed ecosystems (Hämäläinen et al., 2017) since they can effectively disperse most of the seeds contained in their prey.

The gray fox and the coati, had short retention times to disperse the seeds in a maximum time of 21 h while, in other studies with medium‐sized mammals, retention times of no more than 10 h were obtained (Tsuji et al., 2011; Varela & Bucher, 2006; Zhou et al., 2008). In turn, Graae et al. (2004) determined that the arctic fox presents an average seed retention of 44–48 h, which is a much longer period than our results suggest. Likewise, the seed retention time in rabbits did not exceed 24 h, which is similar to that indicated by Cosyns et al. (2005), who observed a retention time of 12 h. The variation in retention times in our study and in previous studies may be the result of the species of seed offered and the other elements in the animals' diets. However, it is essential to determine the retention time of the seeds in the intestines of the animals because it is an important stage in dispersal and can influence the distance the seeds can travel through the landscape (Tsuji et al., 2011). Diploendozoochory by felines produced longer retention times than endozoochory, with averages ranging from 30 h to almost 60 h. Although these represent very long times for seed retention, Pedrosa et al. (2019) also reported long average retention times of 70 h in seeds dispersed by wild boar (*Sus scrofa*), while for another group of vertebrates, such as snakes, their retention average is much longer since the seeds can remain between 6 and 12 days in the digestive tract (Schuett et al., 2022). In particular, the cougar presented very long retention times of up to 96 h, so they can effectively promote the dispersal of seeds over considerable distances (Home range: 83 ± 10.3 km^2^) from the mother plant (Nuñez‐Perez & Miller, 2019; Pedrosa et al., 2019; Varela & Bucher, 2006).

Although the results were not statistically significant, seeds retained in the tracts of most of the mammals maintained a viability equal to or greater than those of the canopy. The passage of the seeds in the tracts did not, therefore, affect viability, except in seeds sourced from the rabbits because of the damage caused by mastication. In their study of arctic foxes, Graae et al. (2004) suggested that seeds must be defecated within 12 h to remain viable; however, our results showed that the gray fox and coati, with seed retention times greater than 18 h, still excreted viable seeds. This is similar to the findings of Rubalcava‐Castillo et al. (2020), who reported that the gray fox and ringtail (*Bassariscus astutus*, from the same family as the coati, *Procyonidae*) also managed to maintain seed viability in the species *J. deppeana*, suggesting that this plant species is adapted to dispersal by endozoochory. The effect of diploendozoochory on the viability of seeds dispersed by the bobcat and cougar showed the highest percentages of viability, exceedingly even those in endozoochory. This result is similar to that reported by Rubalcava‐Castillo et al. (2021), who suggested that seeds remain viable despite passage through the digestive tract of the bobcat. This further confirms the importance of these carnivores in the process of ecosystem regeneration since the seeds remain viable during their passage in the digestive tracts for subsequent dispersal. However, the diploendozoochory process can have different effects on viability depending on the animal and plant species involved, as indicated by Nogales et al. (2015), where dispersal by the lizard (*Gallotia*) and its predator, the wildcat (*Felis*), showed a decrease in the viability in the seeds of two plant species (*Plocama pendula* and *Rubia fruticose*).

The seed testas are external barriers that protect the embryos from the external environment. The thickness of the testa is, therefore, essential for surviving passage through the intestine in animals and for breaking dormancy (Jaganathan et al., 2016). Nevertheless, a clear relationship has not been found between the physical characteristics of the seeds, such as thickness, and their retention times during the dispersal process (Graae et al., 2004; Varela & Bucher, 2006; Willson, 1989). Our results using the SEM showed that the testa thickness in *J. deppeana* seeds dispersed by most mammals did not influence the levels of wear, and there was no decrease measured in the thickness of the testas during passage through the digestive tracts of animals. This differs from the findings of several previous studies in which the authors concluded that exposure of the seeds to this digestive effect can cause changes in the internal and external structure of the testa, generally acting to decrease its thickness (Nogales et al., 2007, 2015; Traveset et al., 2001). Our study suggests the opposite of these observations, recording an increase in the thickness of the testas in the seeds from all mammals, except the rabbits. In the coati through endozoochory, the seed increased until reaching 1105 μm and, in the bobcat through diploendozoochory, the testa increased to 1141 μm. These measurements are much thicker than those mentioned by Venier et al. (2012), who reported a decrease in the thickness of the seeds of *Acacia aroma* (491 μm) when simulating their passage through cattle. The anomaly of the increased thickness of the testas found in our model coincides with that recently reported by Rubalcava‐Castillo et al. (2021), who also recorded an increase in the thickness of the seed testas of *Juniperus* sp. taken from scats of different mammals in temperate forests, and the authors agree that the increase in thickness may be the result of the prolonged time of seed retention in the digestive tracts, as previously verified. When in contact with fluids in the animal's intestine, the seeds could absorb these liquids, causing swelling such as that reported by Varela and Bucher (2006), who indicated that seeds of the *A. aroma* plant species dispersed by foxes showed signs of hydration. This can be an important factor in viability since, when seeds are dispersed with hydration, they can retain moisture, which protects them from viability loss due to drying out (Zavala‐Chávez, 2004).

In response to the long periods of seed retention in endozoochory and diploendozoochory, the testas presented cracks, holes, and removal of protective external layers on the surfaces, which can benefit the seed by conferring characteristics of permeability for the entry of essential elements (water, light, oxygen) for germination (Costea et al., 2016; Schaumann & Heinken, 2002). However, depending on the plant species dispersed, there may be negative aspects, since prolonged exposure of seeds to animal gastrointestinal enzymes may act to reduce germination (Cypher & Cypher, 1999).

The effect of double digestion on seed attrition seen in this experimental evaluation is probably overestimated compared to the typical diploendozoochory that occurs in wildlife because, although in our study seeds pass through the entire digestive tract of both herbivores and carnivores, this is unlikely to happen unless the carnivores eventually consume lagomorph feces that contain seeds, which has not been proven so far. However, the experiment was designed in this way to be conducted ethically by avoiding the need to sacrifice the rabbits. Nevertheless, our results show the real effects of endozoochory and diploendozoochory on retention times and seed wear (scarification) with an emphasis on the predators to demonstrate that they can provide an ecosystem service through scarification and seed dispersal.

## 5 CONCLUSIONS

The evaluation carried out in this study highlights the participation of fox and coati (endozoochory), and bobcat and cougar (diploendozoochory), as an important component of seed dispersal because of the high percentage of dispersed (recovered) seeds and the variable retention times of the seeds. Our results from an evaluation conducted with animals in captivity suggest that these endozoochorous and diploendozoochorous mammalian species seem to contribute to dispersal potential by keeping the seeds of *J. deppeana* viable. These seeds also present adaptive characteristics in the testa to resist digestive scarification.

## AUTHOR CONTRIBUTIONS

**Fabin Alejandro Rubalcava‐Castillo:** Conceptualization (lead); data curation (lead); formal analysis (lead); investigation (lead); methodology (lead); project administration (lead); software (lead); supervision (lead); validation (lead); visualization (lead); writing – original draft (lead); writing – review and editing (lead). **Arturo Gerardo Valdivia‐Flores:** Conceptualization (supporting); data curation (equal); formal analysis (supporting); funding acquisition (lead); investigation (supporting); methodology (supporting); project administration (supporting); resources (lead); software (lead); supervision (supporting); validation (equal); visualization (supporting); writing – original draft (equal); writing – review and editing (equal). **Jos de Jesus Luna‐Ruiz:** Formal analysis (supporting); funding acquisition (supporting); methodology (supporting); project administration (supporting); resources (supporting); supervision (supporting); validation (supporting); writing – original draft (equal); writing – review and editing (equal). **Luis Ignacio Iguez‐Dvalos:** Conceptualization (supporting); investigation (supporting); project administration (supporting); supervision (supporting); validation (supporting); writing – review and editing (supporting). **Victor Manuel Martinez‐Calderon:** Conceptualization (supporting); investigation (supporting); project administration (supporting); validation (supporting); visualization (supporting); writing – review and editing (supporting). **Antonio de Jess Meraz Jimnez:** Conceptualization (supporting); investigation (supporting); project administration (supporting); supervision (supporting); validation (supporting); visualization (supporting); writing – review and editing (supporting). **Joaqun Sosa Ramrez:** Conceptualization (supporting); funding acquisition (supporting); resources (supporting); validation (supporting); writing – original draft (supporting); writing – review and editing (supporting).

## FUNDING INFORMATION

Universidad Autónoma de Aguascalientes, Departamento de Ciencias Veterinarias, No. de Proyecto: PIP/SA22‐2; Universidad Autónoma de Aguascalientes, Departamento de Ciencias Agronómicas/UAAUCMEXUS‐CONACYT, No. de Proyecto: PIAgRN‐18‐1; Refugio de Fauna Silvestre, CEAR Rodolfo Landeros Gallegos.

## CONFLICT OF INTEREST STATEMENT

No authors disclose any conflict of interest.

## Data Availability

Data are available from the Dryad Digital Repository (https://doi.org/10.5061/dryad.pnvx0k6td).
